# Discovery of Dual ETA/ETB Receptor Antagonists from Traditional Chinese Herbs through *in Silico* and *in Vitro* Screening

**DOI:** 10.3390/ijms17030389

**Published:** 2016-03-16

**Authors:** Xing Wang, Yuxin Zhang, Qing Liu, Zhixin Ai, Yanling Zhang, Yuhong Xiang, Yanjiang Qiao

**Affiliations:** 1 Beijing Key Lab of Traditional Chinese Medicine (TCM) Collateral Disease Theory Research, School of Traditional Chinese Medicine, Capital Medical University, Beijing 100069, China; wangxing@ccmu.edu.cn (X.W.); azxccmu@163.com (Z.A.); 2 Key Laboratory of TCM-Information Engineer of State Administration of TCM, School of Chinese Materia Medica, Beijing University of Chinese Medicine, Beijing 100102, China; zhangyuxinwjzy@163.com (Y.Z.); sdliuqing17@tom.com (Q.L.); collean_zhang@163.com (Y.Z.); 3 Department of Chemistry, Capital Normal University, Beijing 100069, China; cnuxiangyh@163.com

**Keywords:** dual ETA/ETB receptor, pharmacophore, molecular docking, aristolochic acid A, bioassay evaluation

## Abstract

Endothelin-1 receptors (ETAR and ETBR) act as a pivotal regulator in the biological effects of ET-1 and represent a potential drug target for the treatment of multiple cardiovascular diseases. The purpose of the study is to discover dual ETA/ETB receptor antagonists from traditional Chinese herbs. Ligand- and structure-based virtual screening was performed to screen an in-house database of traditional Chinese herbs, followed by a series of *in vitro* bioassay evaluation. Aristolochic acid A (AAA) was first confirmed to be a dual ETA/ETB receptor antagonist based intracellular calcium influx assay and impedance-based assay. Dose-response curves showed that AAA can block both ETAR and ETBR with IC_50_ of 7.91 and 7.40 μM, respectively. Target specificity and cytotoxicity bioassay proved that AAA is a selective dual ETA/ETB receptor antagonist and has no significant cytotoxicity on HEK293/ETAR and HEK293/ETBR cells within 24 h. It is a feasible and effective approach to discover bioactive compounds from traditional Chinese herbs using *in silico* screening combined with *in vitro* bioassay evaluation. The structural characteristic of AAA for its activity was especially interpreted, which could provide valuable reference for the further structural modification of AAA.

## 1. Introduction

Endothelin (ET), first described by Yanagisawa in 1988 [1], is a 21-amino acid peptide with strong vasoconstrictor and mitogenic activities [2,3,4,5]. ET-1 is the most abundant endothelin and plays an important role in the cardiovascular system and cardiovascular diseases, such as regulating vascular tone, cardiac contractility, water hemostasis and the production of rennin and aldosterone [6,7]. The biological effects of ET-1 are mediated by two G protein-coupled receptors, ETA receptor (ETAR) and ETB receptor (ETBR) [8,9]. ETAR promotes vasoconstriction, cell growth, adhesion and thrombosis, while ETBR promotes vasodilation [10,11,12]. Endothelin receptor antagonists have been developed for the treatment of multiple cardiovascular diseases, such as hypertension and systemic sclerosis [10,13,14,15]. Moreover, they also show therapeutic action for glaucoma [16], preeclampsia [17], diabetic kidney disease [18] and ovarian tumors [19]. Endothelin receptors have been considered to be potential target in the clinical setting [20]. Recent studies reported that dual ETA/ETB receptors antagonists have efficient activity in treatment of pulmonary arterial hypertension and improving cardiac performance for patients with Eisenmenger’s syndrome [21,22,23].

Guanxin Suhe Pill (GXSHP) is a famous Chinese prescription medicine used to treat acute myocardial ischemia, angina pectoris and myocardial infarction in China [24]. GXSHP is composed of five Traditional Chinese herbs, *i.e.*, *Liquidambar orientalis*, *Dryobalanops aromatic*, *Boswellia carterii*, *Santalum album* and *Aristolochia debilis*. Previous studies showed that GXSHP could significantly increase the production of nitric oxide (NO), enhance the activity of superoxide dismutase (SOD), decrease the contents of malondialdehyde (MDA), glutamate oxaloacetate transaminase (GOT) and reduce the positive rate of Electrocardiogram (ECG) in the acute myocardial ischemia rats model [24]. Thus, it has been successfully used to treat angina pectoris and myocardial infarction caused by coronary heart disease in China.

To identify natural dual ETA/ETB receptor antagonist, ligand-based and structure-based virtual screening was performed to search the herbal ingredients database of GXSHP. Then, the potential active hits were submitted to ETA/ETB receptor antagonism test based on intracellular calcium influx assay and impedance-based assay. Finally, the ETA/ETB receptor antagonistic active ingredients were further evaluated for its target specificity against other G protein–coupled receptors (GPCRs) and cytotoxicity against HEK293/ETAR and HEK293/ETBR cells. The strategy used in this work is efficient to screen large herbal databases for dual ETA/ETB receptor antagonists, and it will play an important role to rationally identify lead compounds from natural products.

## 2. Results

### 2.1. Pharmacophore-Based Virtual Screening

**Figure 1:**
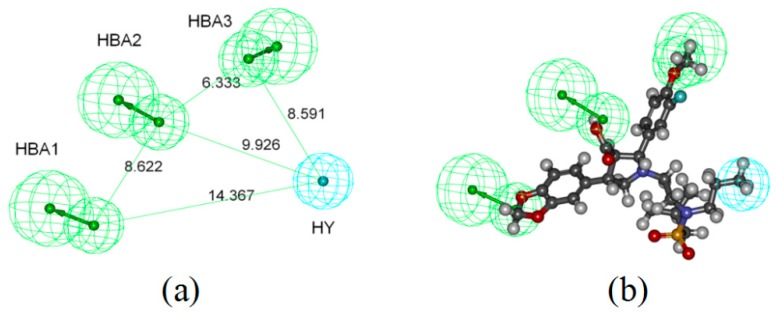
Pharmacophore model of dual ETA/ETB receptor antagonists (**a**) and its overlay on CHEMBL323055 (**b**). The numbers represent the distance between each two pharmacophore features. The arrows represent the direction of hydrogen bond groups. Gray, white, red, blue and yellow spheres represents carbon, hydrogen, oxygen, nitrogen and sulfur atoms, respectively.

To identify dual ETA/ETB receptor antagonists, ligand-based virtual screening on the chemical database of GXSHP was performed using Common Feature Pharmacophore Generation protocol in Discovery Studio v3.5. The top ten pharmacophore models generated (Table S1) were evaluated using the built-in parameters (Table 1). Model_4 (Figure 1a), with the highest comprehensive appraisal index (CAI) [25], was considered to be the best model, which can identify active compounds and exclude inactive compounds comprehensively. Model_4 contains three H-bond acceptors (marked with Green) and one hydrophobic group (marked with Cyan). The best active compound (CHEMBL323055) could map all features of Model_4, with a fit value of 4.46 (Figure 1b). Model_4 was used as a 3D query to screen the chemical database of GXSHP, resulting in a hit list of 17 compounds (Table 2).

### 2.2. Molecular Docking-Based Virtual Screening

#### 2.2.1. Homology Modeling

A high level of sequence identity should guarantee a more accurate alignment between the target sequence and template structure. Here, the crystal structure of the human kappa opioid receptor (PDB ID 4DJH [26]) in complex with JDTic ((3*R*)-1,2,3,4-tetrahydro-7-hydroxy-*N*-[(1*S*)-1-[[(3*R*,4*R*)-4-(3-hydroxyphenyl)-3,4-dimethyl-1-piperidinyl]methyl]-2-methylpropyl]-3-isoquinolinecarboxamide), determined at the resolution of 2.9 Å, shows the highest total score with maximum sequence homology and less *E*-value for both ETAR and ETBR. Thus, it was selected as the template to establish the 3D structures of ETAR and ETBR, respectively. The sequence alignment of the template protein with ETAR and ETBR were obtained using Modeller 4.0 software [27]. The identification parameters of ETAR and ETBR compared to the template were shown in Table 3.

Quality and reliability of the structure was checked by several structure assessment methods including Ramachandram plots, Z-score and ERRAT. ERRAT is a program for verifying protein structures determined by crystallography. The result of the Ramachandran plot of ETAR showed that 81.1% of all residues located in the most favored regions, 14.0% are in additionally allowed regions and 3.1% are in generously allowed regions (Figure S1a). Ramachandran plot of ETBR showed that 79.7% of all residues located in the most favored regions, 14.9% are in additionally allowed regions and 2.8% are in generously allowed regions (Figure S1b). The average, root meam square (RMS) and distribution of Z-scores determined for ETAR and ETBR were show in Figure S2. ERRAT showed overall quality factor of 91.89 for ERAR (Figure S3a) and 91.42 for ERBR (Figure S3b). The Ramachandran plot, Z-scores and ERRAT results confirmed the quality of the homology models, suggesting that the homology model of ETAR and ETBR established could be used for further studies.

Using the Multi-Channel Surfaces module, four and five cavities were generated from the surface of ETAR and ETBR, respectively. Site-directed mutagenesis from the former studies provided an important reference to identify the active sites. Previous studies have confirmed that Tyr129 [28], Lys140 [29], Asp126 and Asp133 [30] played an important role in high-affinity binding to the ETA receptor. For ETB receptor, Asp147 corresponds to the highly conserved aspartate present in TM2 of many GPCRs that has frequently been shown to be crucial for agonist efficacy. In this study, considering the reported key amino acid residues involved in the Surfaces generated, Surface_001 of ETAR and Surface_002 of ETBR, which cover most of the reported key amino acid residues, were selected as the active sites to generate the protomol for molecule docking.

#### 2.2.2. Molecular Docking

Bosentan, a known nonpeptide dual ETA/ETB receptor antagonist [31], was docked into ETAR and ETBR to validate the reliability of the docking protocol. The result showed that bosentan could bind to ETAR via H-bond interaction with Gln72, Thr396 and σ–π interaction with Phy371. For ETB receptor, Ser80, Arg83, Thr84 and Ala385 were the key amino acid residues binding to bosentan through H-bond interactions (Figure S4). The total scores were calculated as 6.54 and 8.58 for ETAR and ETBR, respectively. It indicates that the docking protocol established could reasonably predict the docking mode of known dual ETA/ETB receptor antagonist. All compounds from the 3D chemical database of GXSHP were docked into the active site of ETAR and ETBR using Sulflex-Dock program of SYBYL X-1.2 package. Molecular docking results showed that 17 compounds with docking scores above 5.0 were hit. The docking scores, crash and polar values were shown in Table 4.

### 2.3. Top Scoring Compounds

**Figure 2:**
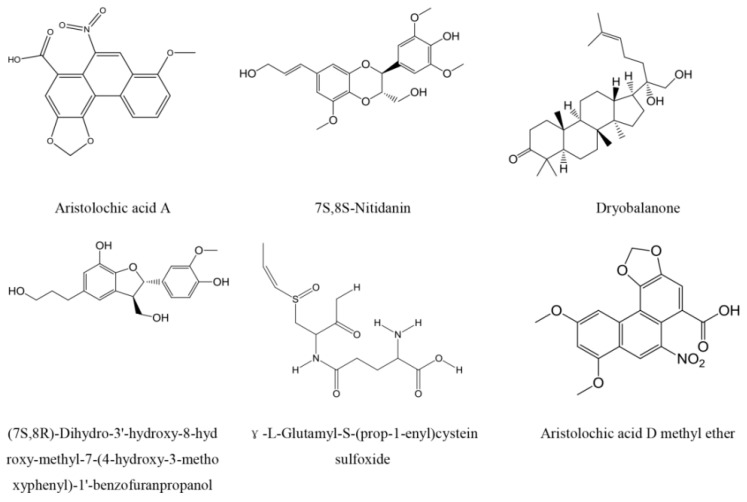
Chemical structure of potential dual ETA/ETB receptor antagonists from GXSHP.

Comparing the ligand- and docking-based virtual screening, six compounds (Figure 2) from GXSHP could both fit to the pharmacophore model well and provide reasonable docking results. They were aristolochic acid A, 7*S*,8*S*-Nitidanin, Dryobalanone, (7*S*,8*R*)-Dihydro-3′-hydroxy-8-hydroxy-methyl-7-(4-hydroxy-3-methoxyphenyl)-1′-benzofuranpropanol, γ-l-Glutamyl-*S*-(prop-1-enyl)cystein sulfoxide and aristolochic acid D methyl ether. Aristolochic acid A is a common compound that contained in many natural herbals, such as *Aristolochia contorta*, *Aristolochia manshuriensis*, *Aristolochia fangchi*, *Aristolochia heterophylla*, *etc.* Previous studies have demonstrated that aristolochic acid A has the pharmacological effect of protecting against infections and inflammation, inhibiting the growth of bacteria [32,33], blocking H_2_O_2_-induced platelet aggregation, suppressing hydroxyl radical induced platelet activation through the arachidonic acid pathway [34,35], and increasing the levels of NO/cyclic guanosine monophosphate (GMP) and cyclic GMP-induced vasodilator-stimulated phosphoprotein phosphorylation [36]. The six compounds were purchased from National Institutes for Food and Drug Control for further *in vitro* assays. The purity of all compounds was over 98% on the basis of HPLC analysis.

### 2.4. ETA/ETB Receptor Antagonism Assay

#### 2.4.1. Intracellular Calcium Mobilization Assay

**Figure 3:**
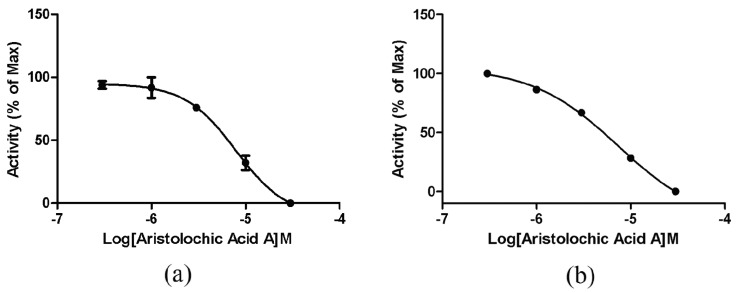
Dose-response curves for aristolochic acid A using calcium influx assay in HEK293/ETAR cells (**a**) and HEK293/ETBR cells (**b**). All error bars indicate SE of the three replicates.

The six selected compounds from virtual screening were assessed at 10 μM for their ability to antagonize ETA/ETB receptor in recombinant cells (human embryonic kidney (HEK)/ETAR and HEK/ETBR cells) using the intracellular calcium mobilization assay. BQ-123 (CAS No. 136553-81-6) [12] and BQ-788 (CAS No. 156161-89-6) [37] were used as reference positive control for ETAR and ETBR assays, respectively. Among the six compounds, only aristolochic acid A was confirmed to be a validated hit with the inhibition rate greater than 50%. The IC_50_ values of aristolochic acid A antagonizing ETAR and ETBR were determined to be 7.91 and 7.40 μM, respectively (Figure 3). The positive control experiment results showed that BQ-123 and BQ-788 could block ETAR and ETBR with IC_50_ of 0.27 and 0.05 nM, respectively (Figure S5), which were very close to the values (IC_50_ = 7.3 nM for BQ-123 and 2.4 nM for BQ-788) reported in the literature [38].

#### 2.4.2. Impedance-Based Assay in Endogenously Expressed Endothelin Receptors Cells

**Figure 4:**
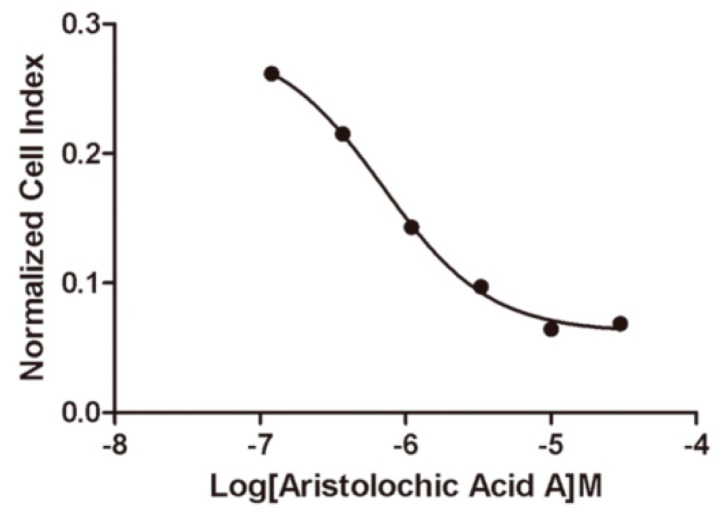
Dose-response curve for aristolochic acid A using impedance-based assay in Hela cells.

To further validate the potency of aristolochic acid A inhibiting the endothelin receptors, impedance-based cells were performed in Hela cells that endogenously expressed both ETA and ETB receptors. The resulting dose-response relationships of aristolochic acid A are plotted by the xCELLigence system. The calculated IC_50_ value of aristolochic acid A inhibiting endothelin receptors is 0.70 μM (Figure 4).

### 2.5. Selectivity of Aristolochic Acid A

**Figure 5:**
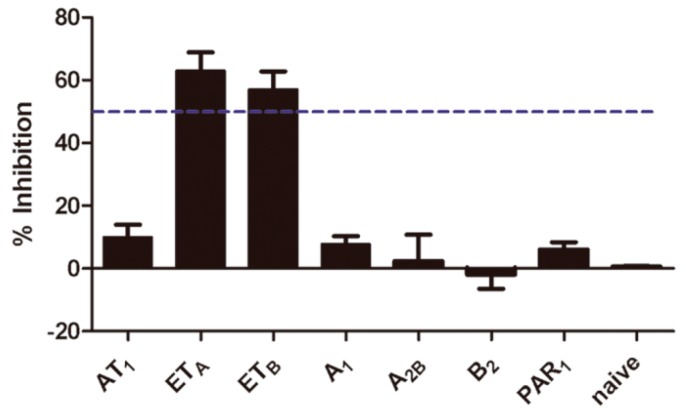
Selectivity assay of aristolochic acid A. All error bars indicate SE of the three replicates. The dash line represents 50% inhibition to the targets.

The selectivity of aristolochic acid A was further assessed by the calcium influx assay against 7 GPCRs, *i.e.*, angiotensin II type 1 receptor (AT_1_), ETA/ETB receptors, adenosine A_1_/A_2_B receptor, B_2_ bradykinin receptor (B_2_) and proteinase-activated receptor 1 (PAR1). Initial screening was conducted at the final concentration of 10 μM. The screen results showed that aristolochic acid A was only active at ETA and ETB receptors in the screening panel, so it was proven to be a selective dual ETA/ETB receptor antagonist (Figure 5).

### 2.6. Compound Cytotoxicity Evaluation

**Figure 6:**
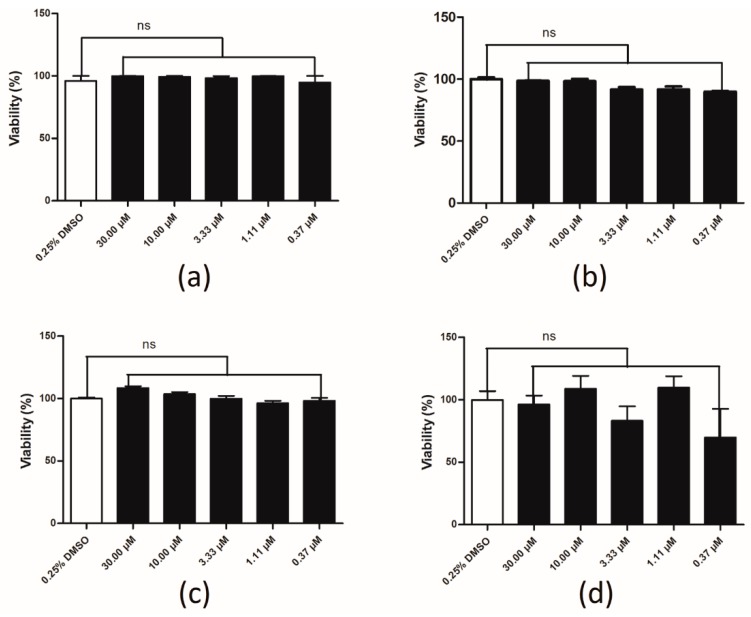
Cytotoxicity evaluation of aristolochic acid A for 1 (**a**), 6 (**b**), 12 (**c**) and 24 (**d**) h. ns mean there were no significant difference between the control group and the AAA groups.

A luciferase coupled adenosine triphosphate (ATP) quantitation assay was used to determine the cytotoxicity of aristolochic acid A. Compounds were incubated in HEK293 cell for 1, 6, 12 and 24 h before luminescence signal measurement. Compared to the control group, aristolochic acid A showed no significant cytotoxicity on HEK293 cell within 24 h (Figure 6). Values are means of replicates of three independent experiments ± standard error.

### 2.7. Binding Mode

**Figure 7:**
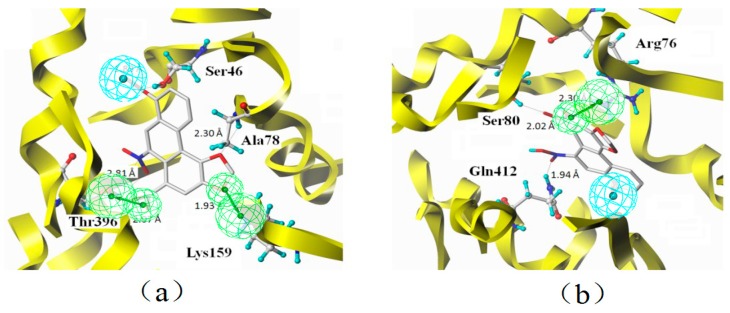
Binding modes between aristolochic acid A and ETAR (**a**) or ETBR (**b**). (The hydrogen bonding interactions are displayed in dotted lines. The green and blue spheres represent hydrogen bond acceptor and hydrophobic groups, respectively.)

Accurate prediction of ligand-protein binding mode can not only shed light on the mechanism of the molecular recognition but also promote drug optimization. The binding mode of aristolochic acid A with ETAR and ETBR was predicted (Figure 7). The docking results implied that Ser46, Ala78, Lys159 and Thr396 of ETAR and Arg76, Ser80 and Gln412 of ETBR are the key amino acid residues binding to aristolochic acid A.

## 3. Discussion

### 3.1. Combinatorial Virtual Screening and in Vitro Bioassay Validation

A systematic combination of virtual screening and *in vitro* bioassay was established to identify dual ETA/ETB receptor antagonists from traditional Chinese herbs. A natural molecule with high potency was identified and experimentally validated. The computational work was performed in less than a month on a single computer. Together with the *in vitro* bioassay validation, the whole process took no more than two months and required significantly less resources than similar drug discovery efforts. This combinational approach is a cost-effective and time saving process in rational questing for target-based active ingredients from natural herbals. The methods established in this study through *in silico* and *in vitro* screening could efficiently used to screen other dual ETA/ETB receptor antagonist. It also helps to interpret the structural characteristics in aristolochic acid A for its activity

### 3.2. Characteristic Analysis of Aristolochic Acid A for Its Activity

The essential characteristic of aristolochic acid A was analyzed through an overlay between pharmacophore features and docking sites. We found that Carboxyl, Nitrogen dioxide and Dioxolane groups are critical to aristolochic acid A’s antagonistic activity. The oxygen atom from the carbonyl group in carboxy group has important hydrogen bonding interaction with Thr396 (ETAR) and Arg76 (ETBR) as a hydrogen bond acceptor. Nitrogen dioxide group could interact with Thr396 (ETAR) and Gln412 (ETBR) through hydrogen bonding interactions. Dioxolane group has hydrogen bonding interaction with Ala78 (ETAR). In addition, the methyl in methoxy group has hydrophobic interaction with the hydrophobic amino acid residues. All the interactions are responsible to the aristolochic acid A’s antagonistic activity against ETAR and ETBR.

### 3.3. Aristolochic Acid A

Aristolochic acid A, as a major active component of medicinal plants from the Aristolochiaceae family of flowering plants, has been widely utilized for medicinal purposes, such as bladder cancer [39]. However, literature studies confirmed that aristolochic acid A can cause nephrotoxicity [40,41], which has been the subject of warnings from several international regulatory agencies. However, the significance of aristolochic acid A as a selective dual ETA/ETB receptor antagonist still can’t be ignored, so it is a valuable and meaningful work to modify the structure of aristolochic acid A to avoid or reduce its toxicity while maintaining its activity.

The main purpose of this study is to discover dual ETA/ETB receptor antagonists from traditional Chinese herbs and investigate the ligand-receptor binding mode. Compound aristolochic acid A was shown to be effective as dual ETA/ETB receptors antagonist and we propose this compound as a starting point for modification and optimization in order to generate clean and safe blockers in the follow-up study. Equally important, *in vivo* studies should be conducted in the further work.

## 4. Materials and Methods

### 4.1. Pharmacophore-Based Virtual Screening

#### 4.1.1. 3D Chemical Database of GXSHP

A total of 582 compounds isolated from *Liquidambar orientalis*, *Dryobalanops aromatic*, *Boswellia carterii*, *Santalum album* and *Aristolochia debilis* were collected from Traditional Chinese Medicine Database (TCMD 2009, Chinese Academy of Sciences). All the structures were extracted and saved in Mol2 format. The chemical structures were converted to 3D conformers using CONCORD module in SYBYL X-1.2 (Tripos Inc., St. Louis, MO, USA). Then, the structures were checked, energy optimized by Tripos force field and stored as a 3D chemical database of GXSHP.

#### 4.1.2. Generation of Common Feature Pharmacophore Model

**Figure 8:**
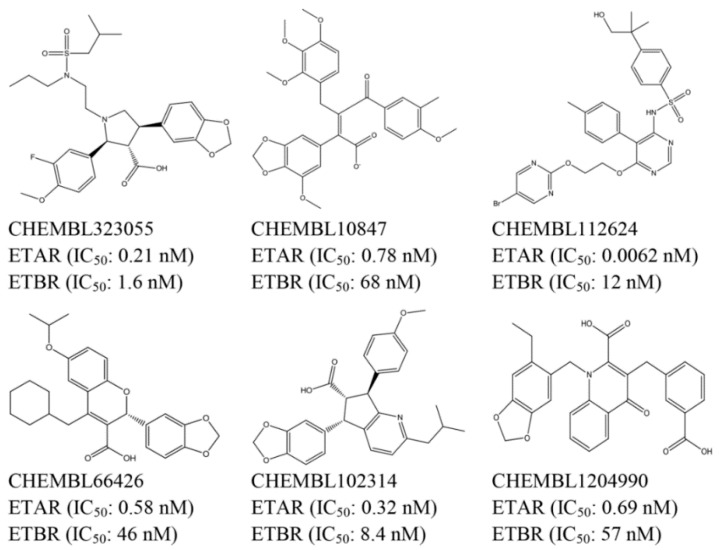
Training set used in pharmacophore generation.

A diverse dataset of 100 experimentally known dual ETA/ETB receptor antagonists were retrieved from published literature [42,43,44,45,46,47,48,49,50,51,52,53]. All the compounds were initially sketched and converted to 3D structures with all proton and MMFF94 charges added using SYBYL X-1.2. To generate the pharmacophore model, six compounds were selected as the training set (Figure 8) according to the following criteria: (a) sharing certain structural diversity; (b) high antagonistic activity in each series; (c) contain similar pharmacophore features to ensure their similar binding models. The other antagonists were used as validation.

Common Feature Pharmacophore Generation protocol in Discovery Studio v3.5 (Accelrys, San Diego, CA, USA) was used to generate the ligand-based pharmacophore models. A principal value of 2 and a maximum omit feature value of 0 were assigned to the six compounds in the training set. Energy minimization optimization was performed with CHARMM force field for all the compounds. Poling algorithm was used to generate a maximum of 255 diverse conformations with the threshold of 20 kcal·mol^−1^ above the calculated lowest energy for each compound in the training set. The conformers were generated using Diverse Conformer Generation protocol running with “best conformer generation” option as available in DS. All the training set compounds associated with their conformations were used to generate the pharmacophore using “common feature pharmacophore generation” in DS. “Feature Mapping” protocol was used to identify common features shared by the training set. As predicted, hydrogen bond acceptor (HBA), hydrogen bond donor (HBD), hydrophobic (HY) and Ring hydrophobic aromatic (RH) features were selected during the pharmacophore generation. Ten possible pharmacophore hypotheses having a different arrangement of constituent features were generated in each hypothesis running. The hypotheses were sorted according to the ranking scores. Redundant hypotheses having the same chemical characteristics and nearly the same distances between these functions were deleted [54].

#### 4.1.3. Pharmacophore Validation and Virtual Screening

The pharmacophore models were validated through an external database of decoy set, consisting of 144 experimentally known dual ETA/ETB receptor antagonists and 340 inactive compounds retrieved from literature. Four parameters (*i.e.*, A%, Y%, N and CAI) proposed in our previous work [55] were calculated to evaluate the performance of the models. D is the number of compounds in the external database while A is the number of active compounds. Ht is the number of hits and Ha is the number of active hits. A% represents the ability identifying active compounds from the external database. Y% represents the proportion of active hits in total hits. N (namely the identified effective index) represents the ability to distinguish active compounds from non-active compounds. CAI, comprehensive appraisal index, was proposed to evaluate of the models comprehensively. The model with the highest CAI was utilized as a 3D query to screen the chemical database of GXSHP.

Virtual screening was performed using the Search 3D Database protocol in DS, with the Minimum Interference Distance set to 1 Å and the Search Method set to Best. All other protocol parameters were maintained as the default settings. Fit value was calculated to indicate the matching degree of each ligand on the pharmacophoric features. A higher fit value suggests a better alignment between ligand’s conformer and pharmacophore model.

### 4.2. Molecular Docking-Based Virtual Screening

#### 4.2.1. Homology Modeling

As the crystal structure of ETAR and ETBR have not yet been resolved, homology modeling was used to construct the three-dimensional structure of ETAR and ETBR, respectively. The sequences of human ETAR (Accession No: AAB20278.1) and ETBR (Accession No: AAB34052.1) were retrieved from the National Center for Biotechnology Information (NCBI) (http://www.ncbi.nlm.nih.gov). The BLAST tool from NCBI [56] was employed to search for the possible template structures from Protein Data Bank (PDB). The protein-protein BLAST algorithm was used. The Scoring Matrix was selected as PAM30. The academic version of MODELER 9v11 [57] was used for homology modeling. Structural features in the template protein were used to derive spatial restraints to generate model protein structures through conjugate gradient and simulated annealing optimization procedures [58]. The regions that were not aligned with identical equivalent parts of the template were optimized further by using a High Level of Optimization during the modeling. After addition of hydrogen atoms, the structure of models was energy-minimized individually using the staged minimization program of SYBYL X-1.2. First, the simplex method was used for 20 cycles before switching to the AMBER FF99 force field for 1000 iterations with the steepest descent (SD) calculation. Then, the conjugated gradient (CG) calculation was implemented until the convergence on the gradient reached 0.05 kcal/(Å·mol).

After the global energy minimization, the stereo chemical quality of the constructed models was assessed using various structure assessment tools such as PROCHECK [59], Z-score and ERRAT [60]. Procheck checks the stereochemical quality of a protein structure by analyzing residue-by-residue geometry and overall structure geometry. This tool was used to determine the Ramachandran plot to assure the quality of the model. The Z-score is indicative of overall model quality and is used to check whether the input structure is within the range of scores typically found for native proteins of similar size. Reliability of the model was further checked by ERRAT, which analyzes the statistics of non-bonded interactions between different atom types and plots the value of the error function *versus* position of a nine-residue sliding window, calculated by a comparison with statistics from highly refined structures. All the structure assessment values were estimated using an online tool SAVES (http://services.mbi.ucla.edu).

#### 4.2.2. Active Site Identification

To identify the ligand binding site, multi-channel surfaces searching method in SYBYL X-1.2 was used to search the cavities on the surface of the protein. Considering the binding site mutagenesis experiments reported from the published literature, the cavity containing active amino acid residues was selected as the active pocket surface to generate protomol for molecular docking. The protomol was generated using the steric hydrophobic group (CH_4_), the hydrogen bond group (C=O), and the hydrogen acceptor (N-H) within 4.5 Å of the active pocket surface.

#### 4.2.3. Molecular Docking

Surflex-Dock, a well-recognized method in the field of molecular docking [61,62], was employed to perform virtual screening and calculate the ligand-receptor interaction. To check the accuracy of the docking program, bosentan was docked into the active site of ETAR and ETBR, respectively. After validation, the compounds from the database of GXSHP were prepared as the following procedure: the structures were checked and the hydrogen atoms were added, the atomic charges were added by Gasteiger–Hückel method, an energy minimization was implemented using the Tripos force-field for 1000 iterations. Then, the optimized compounds were docked into the active site of ETAR and ETBR using default settings, respectively. After each Surflex–Dock run, the best ten docked conformers or poses were sorted in a molecular spreadsheet, and they represent binding affinities in −log 10(Kd) based on surflex-dock scoring function (crash score (also pKd units), polar score, D-score, PMF-score, G-score, ChemSco and CScore) [63]. The common hits through ligand–based and structure-based virtual screening were subjected to the ETA/ETB receptor antagonism assay.

### 4.3. ETA/ETB Receptor Antagonism Assay

#### 4.3.1. Intracellular Calcium Mobilization Assay

HEK/ETAR and HEK/ETBR cells, provided by the Beijing Institute of Genomics in the Chinese Academy of Science, were well-characterized cell lines expressing ETA or ETB receptor. They were plated in 96-well clear-bottom black plates (costar) coated with matrigel (BD) at a density of 30,000 cells per well and cultured overnight in complete media. In the following day, growth media were removed from the cell plates before testing and replaced with 100 μL assay buffer containing a final concentration of 4 μM calcium-sensitive dye Fluo-4-AM (Molecular Probes, Waltham, MA, USA ), pluronic F127 (0.04% in DMSO, Sigma-Aldrich, Beijing, China) and 2.5 mM probenecid (purity ≥ 98%, Sigma-Aldrich) in Hanks’ buffered salt solution (HBSS). Cells were incubated in a humidified atmosphere of 5% CO_2_ at 37 °C for 30 min. The intracellular Ca^2+^ flux was assayed using a fluorescent imaging plate reader (FlexStation II; Molecular Devices, Shanghai, China) to monitor Fluo-4-AM fluorescence in all wells simultaneously (λ excitation = 485 nm, λ emission =525 nm). Cells were challenged with agonist ET-1, and the fluorescence intensity was captured every 1.52 s for 80 s. For antagonists effects test, the cells were preincubated with the test compounds for 10 min prior to calcium-flux measurement. BQ-123 [64] and BQ-788 [65] were used as reference positive control for ETAR and ETBR assays, respectively.

#### 4.3.2. Impedance-Based Assay in Endogenously Expressed Endothelin Receptor Cells

For this study, 200 μL of medium was added to E-Plate L8 to obtain background readings followed by the addition of 300 μL of hela cells suspension. The E-plates containing the indicated initial number of cells were allowed to incubate at room temperature for 30 min before being placed onto xCELLigence System (ACEA Biosciences, Hangzhou, China) in the incubator for continuous recording of impedance as reflected by cell index (CI). The cells were allowed to attach and grow for 18–24 h to reach a stable baseline before the addition of the indicated compounds. For studying antagonist, medium exchange was conducted and growth media was replaced with serum-free Dulbecco’s modified eagle medium (DMEM). After 2 h of equilibrium on the station to establish the baseline CI, the indicated antagonists were gently added to the well, and after 1 h of incubation, the endothelin receptor agonist ET-1 was added at 100 nM. The results were expressed by normalized CI, which are derived from the ratio of CIs after the addition of the compounds.

### 4.4. Compound Specificity Assay

The selectivity of active compounds were further assessed, in which the compound was tested for inhibiting seven GPCRs, *i.e.*, angiotensin II type 1 receptor (AT1), ETA/ETB receptors, adenosine A_1_/A_2_B receptor, B_2_ bradykinin receptor (B_2_) and proteinase-activated receptor 1 (PAR1). HEK293 cells that are stably expressing those GPCRs were separately plated in a 96-well clear-bottom black plate with a density of 40,000 cells per well. The plate was coated with matrigel and incubated at 37 °C overnight. Screens were conducted at a concentration of 10 μM using calcium assay when cells challenged with their selective agonists for antagonist assay. HEK293 cell line was set up as naive group and cells were challenged with 10 μM ATP (purity ≥ 99%, Sigma-Aldrich) as agonist.

### 4.5. Compound Cytotoxicity Evaluation

HEK293/ETAR and HEK293/ETBR cells were seeded at 3.5 × 10^4^ per well into 96-well clear-bottom black plates and incubated in 5% CO_2_ at 37 °C overnight, respectively. Different concentrations (30.00, 10.00, 3.33, 1.11 and 0.37 μM) of the compound were added into the 96-well plates and incubated in 5% CO_2_ at 37 °C for 1, 6, 12 and 24 h. Luminescence was read by Envision 2100 multi-label reader to detect cells’ viability following incubation with CellTiter-Glo reagent for 10 min.

## 5. Conclusions

In summary, a combined computational approach, including pharmacophore model, homology modeling and molecular docking, has been applied to identify dual ETA/ETB receptor antagonists from traditional Chinese herbs, followed by a series of bioassay evaluations. Finally, aristolochic acid A was first identified to be a selective dual ETA/ETB receptor antagonist. Thus, it is a feasible and effective approach to discover other bioactive compounds from traditional Chinese herbs using *in silico* and *in vitro* screening. The interpretation of structural characteristic for aristolochic acid A’s antagonistic activity especially provided useful reference for the further development of aristolochic acid A’s structure modification.

## Figures and Tables

**Table 1:** Assessment results for each pharmacophore model.

Model	Ht ^a^	Ha ^b^	A (%) ^c^	Y (%) ^d^	N ^e^	CAI ^f^
Model_01	188	114	79.17	60.64	2.04	1.61
Model_02	171	100	69.44	58.48	1.97	1.36
Model_03	187	109	75.69	58.29	1.96	1.48
Model_04	206	123	85.42	56.42	2.01	1.71
Model_05	215	122	84.72	56.74	1.91	1.62
Model_06	205	122	84.72	59.51	2.00	1.69
Model_07	218	123	85.42	59.71	1.90	1.62
Model_08	222	121	84.03	54.50	1.83	1.54
Model_09	213	120	83.33	56.34	1.89	1.58
Model_10	210	123	85.42	58.57	1.97	1.68

^a^ Ht is the number of hits; ^b^ Ha is the number of active hits; ^c^ A% represents the ability identifying active compounds from the external database (A% = Ha/A, while A is the number of active compounds in the external database); ^d^ Y% represents the proportion of active hits in total hits (Y% = Ha/Ht); ^e^ N represents the ability to distinguish active compounds from non-active compounds (N = (Ha/Ht)/(A/D), while D is the number of compounds in the external database); ^f^ CAI was proposed to evaluate of the models comprehensively (CAI = N × A%).

**Table 2:** The hits through pharmacophore-based virtual screening.

ID	Fit Value	Name	Source
15630	3.45	7*S*,8*S*-Nitidanin	*Santalum album*
4208	3.39	CPB-53-641-1	*Santalum album*
5636	3.00	(7*S*,8*R*)-Dihydro-3′-hydroxy-8-hydroxy-methyl-7-(4-hydroxy-3-methoxyphenyl)-1′-benzofuranpropanol	*Santalum album*
1853	2.99	Asiatic acid	*Dryobalanops aromatica*
8780	2.99	γ-l-Glutamyl-*S*-(prop-1-enyl)cystein sulfoxide	*Santalum album*
13843	2.96	7-Methoxy-aristolochiac acid	*Aristolochia debilis*
9799	2.94	7-Hydroxy-aristolochic acid A	*Aristolochia debilis*
1714	2.93	Aristolochic acid D methyl ether	*Aristolochia debilis*
1713	2.92	Aristolochic acid A	*Aristolochia contorta*
6610	2.92	Dryobalanone	*Dryobalanops aromatica*
4814	2.89	Debilic acid	*Aristolochia debilis*
7008	2.89	12-Epirockogenin	*Santalum album*
2568	2.77	β-Boswellic acid	*Boswellia carterii*
10341	2.74	3α-Hydroxy-lup-20(29)-en-24-oic acid	*Boswellia carterii*
2567	2.72	α-Boswellic acid	*Boswellia carterii*
12158	2.61	Kapurol	*Dryobalanops aromatica*
1618	2.60	Arbutin	*Aristolochia debilis*

**Table 3:** The identification parameters of ETAR and ETBR compared to the template.

Target	Template	Max Score ^a^	Total Score ^b^	Query Cover ^c^	*E* Value ^d^	Max Identify
ETAR	4DJH_A	71.9	118	63%	1 × 10^−12^	32%
ETBR	4DJH_A	73.2	118	70%	5 × 10^−13^	31%

^a^ Max score means the score of single best aligned sequence; ^b^ Total score means the sum of scores of all aligned sequences; ^c^ Query cover means the percent of query sequence that is aligned; ^d^ *E* Value means the number of matches with same score expected by chance. Typically, *E* < 0.05 is required to be considered significant.

**Table 4:** Docking results of compounds from Guanxin Suhe Pill (GXSHP).

ID	Name	Source	ETAR			ETBR		
			Total Score	Crash ^a^	Polar ^b^	Total Score	Crash ^a^	Polar ^b^
21206	Tetrandrine	*Aristolochia debilis*	5.98	−3.80	1.33	9.98	−2.81	1.36
8780	γ-l-Glutamyl-*S*-(prop-1-enyl)cystein sulfoxide	*Santalum album*	5.70	−1.88	1.68	7.03	−1.62	3.79
1713	Aristolochic acid A	*Aristolochia debilis*	6.13	−1.42	0.00	6.69	−0.46	4.01
6610	Dryobalanone	*Dryobalanops aromatica*	6.09	−2.28	0.66	6.46	−2.06	1.42
9621	sym-Homospermidine	*Santalum album*	6.15	−0.96	2.85	6.46	−0.54	3.35
5115	Dendrolasin	*Santalum album*	6.44	−1.42	0.66	6.46	−1.30	0.00
12207	Ketosantalic acid	*Santalum album*	5.39	−0.91	1.06	6.45	−2.67	2.94
7730	β-Farnesene	*Santalum album*	6.30	−1.25	0.00	6.31	−0.89	0.00
19307	β-Santalol	*Santalum album*	6.21	−0.96	1.28	6.26	−0.70	2.59
5636	(7*S*,8*R*)-Dihydro-3′-hydroxy-8-hydroxy-methyl-7-(4-hydroxy-3-methoxyphenyl)-1′-benzofuranpropanol	*Santalum album*	6.76	−2.16	4.73	6.21	−2.06	4.63
20417	Styracin	*Liquidambar orientalis*	6.63	−0.60	1.20	6.16	−0.88	2.09
4208	CPB-53-641-1	*Santalum album*	6.54	−1.05	1.03	5.84	−1.30	4.42
19304	β-Santalic acid	*Santalum album*	5.54	−1.11	2.30	5.82	−0.85	1.93
19306	α-Santalol	*Santalum album*	5.37	−0.89	2.61	5.60	−0.57	2.44
15630	7*S*,8*S*-Nitidanin	*Santalum album*	5.15	−1.55	2.70	5.56	−1.98	1.56
1714	Aristolochic acid D methyl ether	*Aristolochia debilis*	5.11	−0.84	1.12	5.23	−0.73	2.52
2307	9(10)Z,α-trans-Bergamotenol	*Santalum album*	5.96	−1.13	1.93	5.05	−2.80	2.58

^a^ Crash means the degree of inappropriate penetration by the ligand into the protein and of interpenetration (self-clash) between ligand atoms that are separated by rotatable bonds. Crash scores close to 0 are favorable. Negative numbers indicate penetration; ^b^ Polar means the contribution of the polar interactions to the total score. The polar score is useful for excluding docking results that make no hydrogen bonds.

## Supplementary Materials

Supplementary materials can be found at http://www.mdpi.com/1422-0067/17/3/389/s1.

## Abbreviations

The following abbreviations are used in this manuscript:

ET-1: Endothelin-1
ETAR: Endothelin subtype A receptor
ETBR: Endothelin subtype B receptor
AAA: Aristolochic acid A
GXSHP: Guanxin Suhe Pill
DS: Discovery Studio software
AT_1_: Angiotensin II type 1 receptor
A_1_: Adenosine A_1_ receptor
A_2B_: Adenosine A_2B_ receptor
B_2_: Bradykinin receptor B_2_
PAR1: Proteinase-activated receptor 1
TCMD 2009: Traditional Chinese Medicine Database 2009
