# Supplementary material for: Discovery of Dual ETA/ETB Receptor Antagonists from Traditional Chinese Herbs through in Silico and in Vitro Screening
Source: Int J Mol Sci. 2016 Mar 16;17(3):389. doi: 10.3390/ijms17030389 (PMC4813245; doi:10.3390/ijms17030389)
Supplement: Supplementary file 1 [file ijms-17-00389-s001.pdf]

Supplementary Materials: Discovery of Dual ETA/ETB Receptor Antagonists from Traditional Chinese Herbs through *in Silico* and *in Vitro* Screening

Xing Wang [1], Yuxin Zhang [2], Qing Liu [2], Zhixin Ai [1], Yanling Zhang [2], Yuhong Xiang [3] and Yanjiang Qiao [2,*]

Table S1. Generation of the pharmacophore models for dual ETA/ETB receptor antagonists.

Model	Features [a]	Rank Score [b]	Direct Hit	Partial Hit	Max Fit [c]
1	HAAA	69.667	111111	0	4
2	HAAA	69.591	111111	0	4
3	HAAA	69.591	111111	0	4
4	HAAA	69.242	111111	0	4
5	HAAA	68.920	111111	0	4
6	HAAA	68.915	111111	0	4
7	HAAA	68.770	111111	0	4
8	HAAA	68.732	111111	0	4
9	HAAA	68.732	111111	0	4
10	HAAA	68.377	111111	0	4

[a] H represents the hydrophobic group; A represents the hydrogen bond acceptor; [b] A higher rank score means a better-quality pharmacophore model; [c] Max Fit mean the number of pharmacodynamic characteristics that match the moleculars.

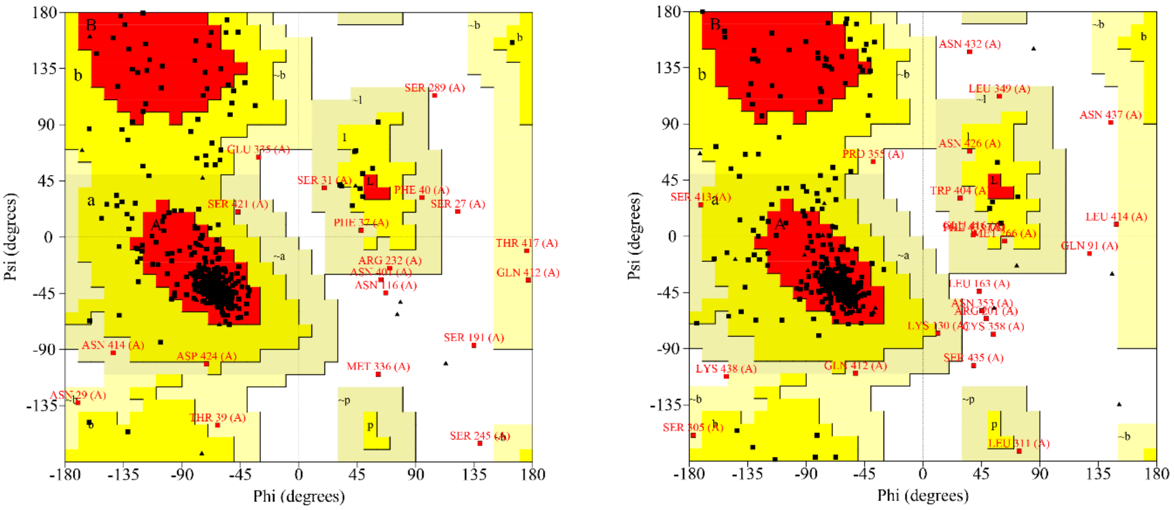

(a) (b)

Figure S1. Ramachandran Plot of modeled ETAR (**a**) and ETBR (**b**). The red, dark yellow and light yellow regions represent the favored, allowed, and "generously allowed" regions, respectively. The other regions are disallowed regions.

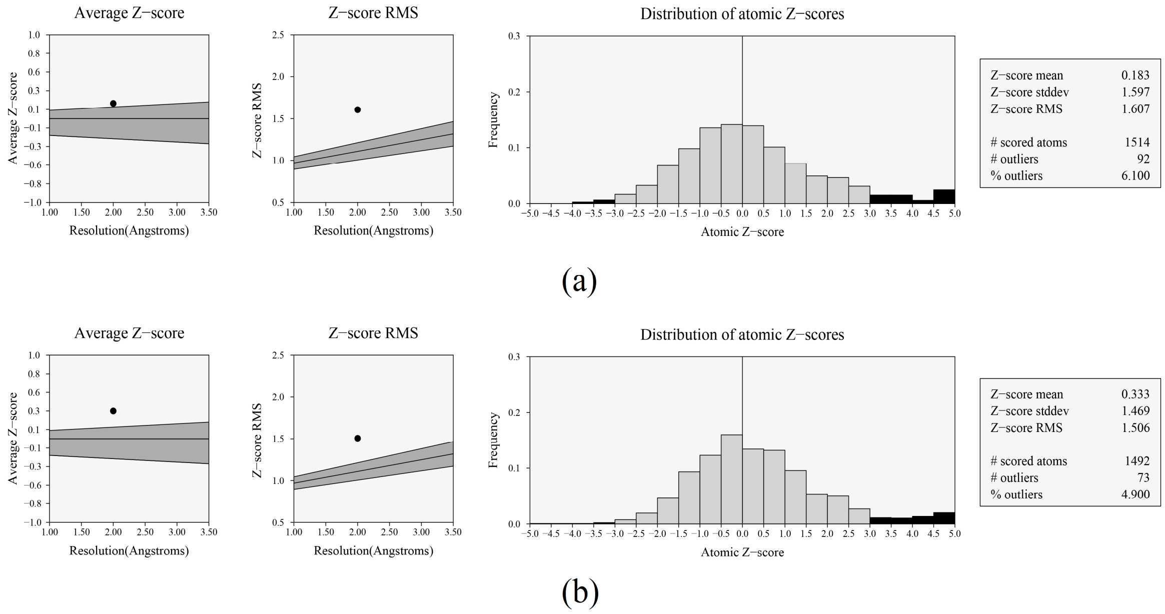

Figure S2. Average, RMS and distribution of Z-scores for ETAR (**a**) and ETBR (**b**).

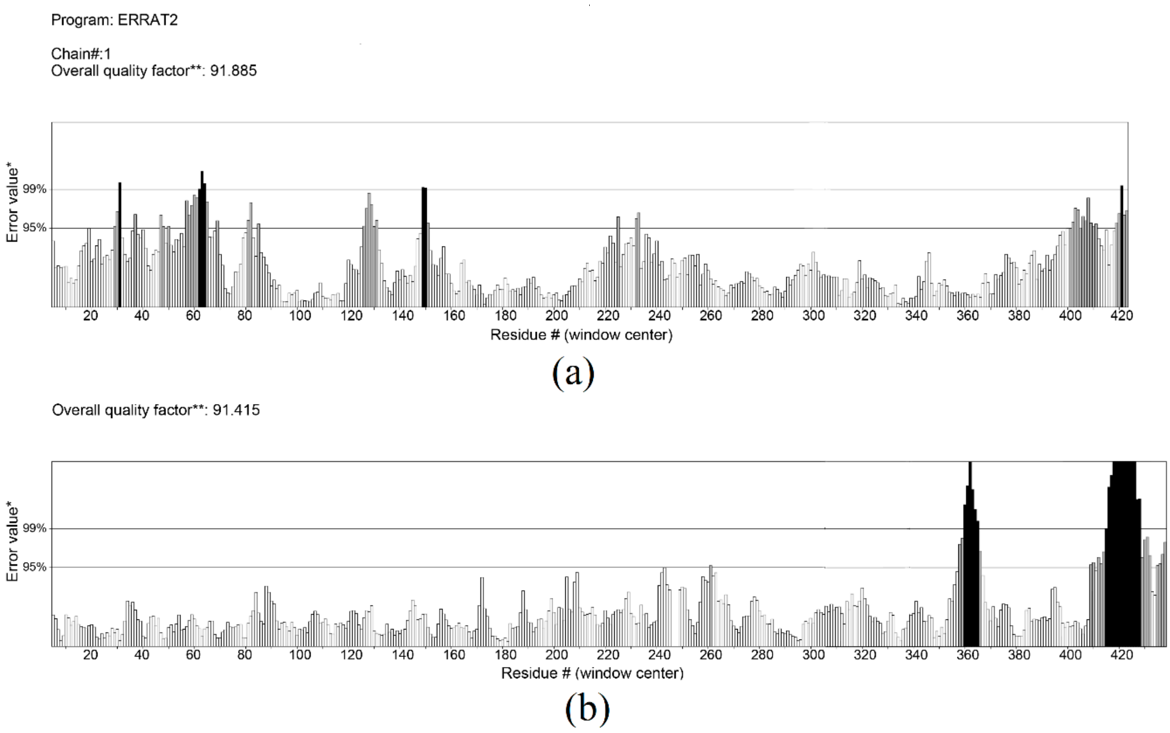

Figure S3. Overall quality factor checked by ERRAT for ETAR (**a**) and ETBR (**b**).

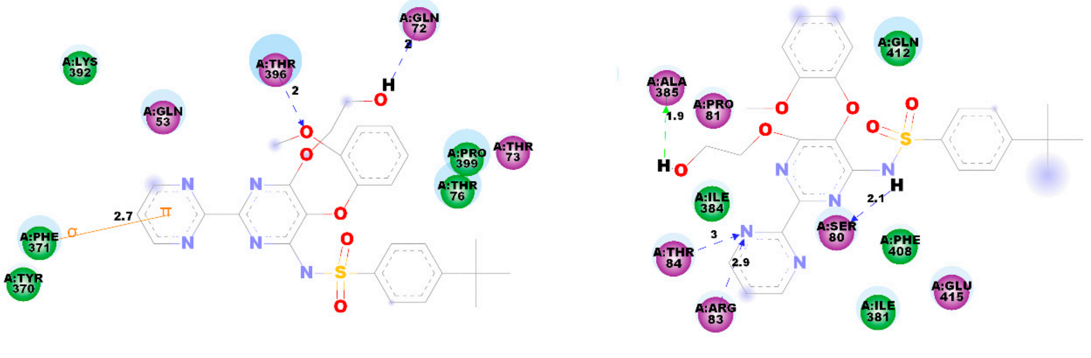

(a) (b)

Figure S4. Binding conformation of bosentan at the active site of ETAR (**a**) and ETBR (**b**). (The green and purple circles represent the key residues of ETAR or ETBR. The dotted lines represent hydrogen bonding interactions and the arrows mean the direction of hydrogen bond interactions.)

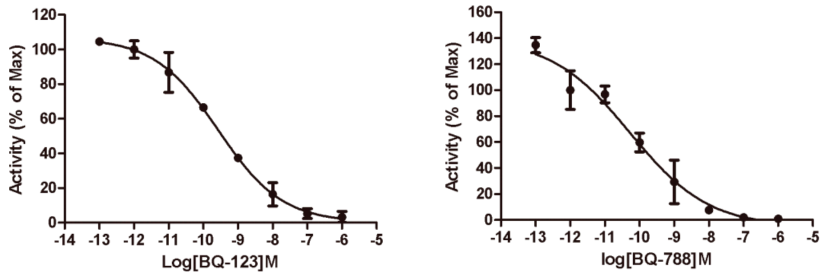

(a) (b)

Figure S5. Dose-response curves using calcium influx assay for BQ-123 in HEK293/ETAR cells (**a**) and BQ-788 in HEK293/ETBR cells (**b**). All error bars indicate SE of the three replicates.
